# Artificial Intelligence–Aided Precision Medicine for COVID-19: Strategic Areas of Research and Development

**DOI:** 10.2196/22453

**Published:** 2021-03-12

**Authors:** Enrico Santus, Nicola Marino, Davide Cirillo, Emmanuele Chersoni, Arnau Montagud, Antonella Santuccione Chadha, Alfonso Valencia, Kevin Hughes, Charlotta Lindvall

**Affiliations:** 1 Division of Decision Science and Advanced Analytics Bayer Pharmaceuticals Whippany, NJ United States; 2 The Women's Brain Project Zurich Switzerland; 3 Department of Medical and Surgical Sciences Università degli Studi di Foggia Foggia Italy; 4 Barcelona Supercomputing Center Barcelona Spain; 5 Department of Chinese and Bilingual Studies The Hong Kong Polytechnic University Hong Kong China (Hong Kong); 6 Biogen International GmbH Baar Switzerland; 7 Institució Catalana de Recerca i Estudis Avançats Barcelona Spain; 8 Massachusetts General Hospital Boston, MA United States; 9 Dana-Farber Cancer Institute Boston, MA United States; 10 Harvard Medical School Boston, MA United States

**Keywords:** COVID-19, SARS-CoV-2, artificial intelligence, personalized medicine, precision medicine, prevention, monitoring, epidemic, literature, public health, pandemic

## Abstract

Artificial intelligence (AI) technologies can play a key role in preventing, detecting, and monitoring epidemics. In this paper, we provide an overview of the recently published literature on the COVID-19 pandemic in four strategic areas: (1) triage, diagnosis, and risk prediction; (2) drug repurposing and development; (3) pharmacogenomics and vaccines; and (4) mining of the medical literature. We highlight how AI-powered health care can enable public health systems to efficiently handle future outbreaks and improve patient outcomes.

## Introduction

The ongoing COVID-19 pandemic has highlighted the fragility of the health care system during unexpected events, testing the endurance of even the top-performing ones [1]. As noted by several scholars, embracing artificial intelligence (AI) for health care optimization and outcome improvement is not an option anymore [2]. Concerning the ongoing COVID-19 pandemic, several studies have highlighted that the timely inclusion of AI-powered technologies would have accelerated the identification of and effective response to COVID-19 outbreaks worldwide. An example is the widely reported algorithm from the Canadian company BlueDot, based on natural language processing (NLP) and machine learning, which forecasted the emerging risk of a virus spread in Hubei province in late December 2019, by screening news reports and airline ticketing [3].

Awareness of the benefits of employing AI to support and manage the COVID-19 crisis and its aftermath is increasing, particularly in the medical and research community. Notable examples of early AI-powered contributions include the discovery of relevant SARS-CoV-2 target proteins by DeepMind’s AlphaFold [4] and the design by Infervision of a computer vision algorithm for the detection of coronavirus pneumonia based on lung images [5].

Benefits do, however, come with technical challenges and related risks that still need to be properly assessed. For example, the absence of transparency and interpretability in AI models obscures the fact that the efficacy of these technologies is not equal across population groups. COVID-19 incidence and outcomes vary according to a large number of individual factors, including age, sex, ethnicity, health status, drug utilization, and others [6]. Sensitizing AI technologies to the diversity of the patient population and ensuring data security [7] is imperative to avoid biased decisions [8-10]. Therefore, a crucial step to obtain robust, trustworthy, and intelligible applications that account for demographic equity is to assess potential biases in the resources used to train AI models for precision medicine [11].

As of today, AI systems are, regrettably, rarely endowed with robustness to class imbalances, such as sex and gender groups [12]. In this regard, sex differences in COVID-19 cases, as well as sex-specific risk factors and socioeconomic burden, have been recently highlighted in a case study by the European Commission [13]. Dataset multidimensionality that can fairly represent the population constitutes one of the main challenges for biobanking and cohort design efforts that collect different axes of health data [14]. In this regard, fair and broad data collection systems are of primary importance. Two essential international references for COVID-19 genomic and medical data are the EMBL-EBI COVID-19 Data Portal [15] and the NIH National COVID Cohort Collaborative (N3C) [16]. The COVID-19 Host Genetics Initiative [17] is an international collaborative undertaking to share resources to investigate the genetic determinants of COVID-19 susceptibility, severity, and outcomes [18]. The Coronavirus Pandemic Epidemiology (COPE) consortium aims to involve experts in the development of a personalized COVID-19 Symptom Tracker mobile app that works as a real-time data capture platform [6], which garnered over 2.8 million users in a few days. Moreover, COVID-19 sex-disaggregated data are collected by Global Health 50/50 [19], an initiative housed at University College London, advocating for gender equity.

Other ethical concerns include life-or-death decisions through risk prediction models, which may help optimize resource allocation in times of scarcity. The application of nonoptimal models may incur the risk of worsening biases and exacerbating disparities for people with serious illnesses and different treatment priorities, potentially causing the reduction in the use of services rather than achieving the best patient care [20]. Nevertheless, the power of prediction models is impressive, and it may play a key role in the future if properly exploited. For instance, a study from Cambridge University [21] shows how the use of secure AI operating on anonymized COVID-19 data can accurately predict the patient journey, allowing an optimal allocation of resources and enabling well-informed and personalized health care decision-making. This is a particularly important point, especially considering the difficulty in managing the increasing need for intensive care units (ICUs) during the COVID-19 pandemic peak [22,23].

The way the AI systems will be exploited is probably the most delicate topic in this adoption process, particularly if we refer to the decisional independence of the medical staff. As humans, in fact, clinicians are also affected by numerous cognitive biases, including the *confirmation bias*, which may lead them to give excessive importance to the evidence supporting automated prediction (eg, risk prediction, diagnosis, and treatment suggestion) and ignore evidence that refutes it [8,24].

Despite the abovementioned concerns, there are numerous success stories in the adoption of risk prediction models. For example, Duke University adopted a system called Sepsis Watch that identifies in advance the inflammation leading to sepsis—one of the leading causes of hospital deaths. Within two years from the tool introduction, the number of sepsis-induced patients drastically decreased [25], thanks to three key elements: (1) adaptation of the predictive model to a highly specific context; (2) scalability through integration with hospital workflows; and (3) the adopted user experience–based approach, which places clinicians and health care professionals at the center of the software development process, adhering with the human-in-the-loop paradigm [26,27].

The COVID-19 crisis is accelerating anticipated changes towards a stronger collaboration between computer science and medicine. In particular, the crisis has exposed the need for increased scrutiny of the relationship between AI and patients as well as health care personnel under the lens of human and emotional needs, as demonstrated by the surge of mental health consequences of the pandemic [28] and the growing development of AI-based mental health apps and related digital tools [29]. Such aspects, together with others related to general data access and the use of AI for disease outcome prediction, are fueling the current debate about the convergence of AI and medicine [30,31] and the actionable realization of AI-powered innovations to bridge the gap between technological research and medical practice, including applications in medical triage and advice, diagnostics and risk-adjusted paneling, population health management, and digital devices integration [32]. Concerning this aspect, it is important to mention the recent publication of guidelines for the rigorous and transparent adoption of AI in the clinical practice: CONSORT-AI (Consolidated Standards of Reporting Trials–Artificial Intelligence) [33] and SPIRIT-AI (Standard Protocol Items: Recommendations for Interventional Trials–Artificial Intelligence) [34].

**Figure 1:**
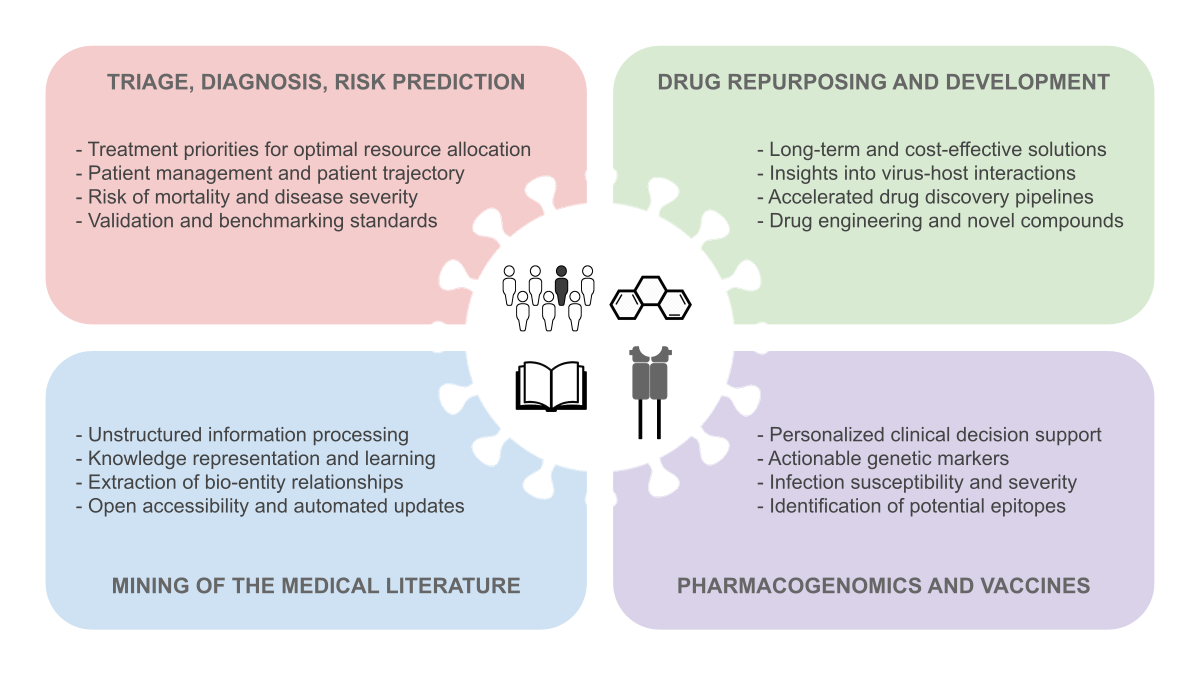
Main strategic areas of research and development for the realization of artificial intelligence (AI) to fight COVID-19: (1) triage, diagnosis, and risk prediction; (2) drug repurposing and development; (3) pharmacogenomics and vaccines; and (4) mining of the medical literature. The text within the four panels enlists the advantages and actionable solutions exhibited by the AI-aided precision medicine approaches surveyed in this work.

Translating patient data to successful therapies is the major objective of implementing AI for health [35], especially in times of a pandemic crisis, with the ultimate goal of achieving a successful bench-to-bedside model for better clinical decision-making [36,37]. In this work, we review some major examples of what AI has achieved during the COVID-19 pandemic and the challenges that this technology and the medical community are currently facing in four main strategic areas of research and development (Figure 1): (1) triage, diagnosis, and risk prediction; (2) drug repurposing and development; (3) pharmacogenomics and vaccines; (4) mining of the medical literature.

## Triage, Diagnosis, and Risk Prediction

AI has been applied to determine treatment priorities in patients with COVID-19 or triage and to better allocate limited resources. A group of researchers at the General Hospital of the People’s Liberation Army (PLAGH), Beijing, China, has developed an online triage tool model [38] to manage suspected COVID-19 pneumonia in adult patients with fever [39]. Using clinical symptoms, routine laboratory tests, and other clinical information available at admission (eg, clinical features), they trained a model based on logistic regression with the least absolute shrinkage and selection operator (LASSO), obtaining an area under the receiver operating characteristic curve (AUROC) of 0.841 (100% sensitivity and 72.7% specificity). Based on data from two hospitals in Wenzhou, Zhejiang, China, another study group recently used an entropy-based feature selection approach: they modeled combinations of clinical features that could identify initial presentation patients who are at a higher risk of developing severe illness, with an accuracy of 80% [40]. Their results show that mildly elevated alanine aminotransferase levels, the presence of myalgias (body aches), and an elevated hemoglobin level (red blood cells), in this order, are predictive of the later development of acute respiratory distress syndrome.

A thorough study on risk prediction was carried out at the University of Cambridge based on the development of a proof of concept system to model the full patient journey through risk prediction models [21]. By identifying the risk of mortality and ICU/ventilator need, the system aims at enabling doctors to answer questions such as: Which patients are most likely to need ventilators within a week? How many free ICU beds in the hospital are we likely to have in a week from now? Which of two patients will get more benefits from going on a ventilator today? The predictive models showed accuracies ranging from 77% for ventilator need to 83% for ICU admission and 87% for mortality.

Risk prediction models are not new to the AI-aided health care approach. They have already been successfully utilized for tasks such as predicting the risk of developing cancer [41,42] and identifying which patients are likely to benefit from heart-related procedures [43]. However, the COVID-19 crisis has accelerated the utilization of such models. In a recent study, Wynants and collaborators [44] screened 14,217 published titles about the pandemic from PubMed and Embase (Ovid, arXiv, medRxiv, and bioRxiv), finding over 107 studies describing 145 prediction models. Among them, 4 models aimed to identify people at risk and 50, to predict the mortality risk, progression to severe disease, ICU admission, ventilation, intubation, or length of hospital stay. These models not only provide interesting results but also inform about the most valuable predictors, such as age, body temperature, lymphocyte count, and lung imaging features. Despite this, these models cannot be directly applied in the clinical setting without further validation, in order to guarantee data and experiment transparency and robustness, together with decision interpretability and model generalizability.

The remaining 91 models from this study were dedicated to the diagnosis of COVID-19, 60 of which exploited medical imaging. This number clearly shows that diagnosis is another important field for the application of AI techniques [45], with digital pathology exhibiting high effectiveness. In particular, convolutional neural networks (CNNs) have been supporting radiologists in their expert decisions [46]. In a recent study, a CNN was trained to automatically learn patterns related to COVID-19 (ie, ground-glass opacities, multifocal patchy consolidation, and/or interstitial changes with a predominantly peripheral distribution), achieving an AUROC of 0.996 (98.2% sensitivity and 92.2% specificity) and outperforming the reverse-transcription polymerase chain reaction, which also suffers from a significant time lag. In addition to accuracy, these approaches put the speed of the diagnosis on the table: CNNs can analyze up to 500 images in a few seconds, whereas radiologists would need hours to complete the same task.

Although chest computed tomography (CT) scans represent a commonly exploited source of information to train AI to rule out SARS-CoV-2 infection, the rapid detection of patients with COVID-19 can greatly benefit from learning approaches that utilize heterogeneous types of data. In this regard, it is crucial to consider the importance of training CNNs in a correct gender balance in medical imaging datasets to avoid producing distorted classifications for assisted diagnosis [12]. Moreover, it is crucial to rely on high-quality benchmarking and robust validation strategies to assess the generalization of the model to other datasets and populations [47,48].

Indeed, AI can exploit multidimensional data, including the series of epidemiological, clinical, biological, and radiological criteria defined by the World Health Organization [49]. In a collaboration between researchers at hospitals in China and in the USA, CNN and other machine learning methods (eg, support vector machine, random forest, and neural networks) have been used to model and integrate CT scans and clinical information for diagnostic purposes [45]. The joint model that uses both information sources achieved a 0.92 AUROC (84.3% sensitivity and 82.8% specificity), outperforming the individual models. Moreover, the models allowed the identification of age, viral exposure, fever, cough, cough with sputum, and white blood cell counts as the main features associated with SARS-CoV-2 infection status.

Recently, the National Institute of Biomedical Imaging and Bioengineering has launched the Medical Imaging and Data Resource Center with the goal of coupling AI and medical imaging for COVID-19 early detection and personalized therapies [50].

AI has also been utilized to identify patients at higher risk of mortality. Researchers at the Tongji Hospital, Wuhan, China, have screened electronic health records of 375 discharged patients to use clinical measurements as features and have trained a gradient-boosted decision tree model to predict mortality risk [51]. The accuracy of the system was 93%. Its utilization would make it possible for physicians to immediately identify critical cases and act accordingly. The model was also able to detect three key clinical features, that is, lactic dehydrogenase, lymphocyte count, and high-sensitivity C-reactive protein.

## Drug Repurposing and Development

Although triage, diagnosis, and risk prediction are three of the most relevant tasks that AI has helped with during the peaks of the pandemic, other objectives are currently being addressed for long-term solutions. Among them are target selection for drug repurposing [52] and approaches for drug development, including de novo drug design [53].

Drug repurposing comprises identifying existing drugs that could effectively act on proteins targeted by the virus. Recently, 332 high-confidence SARS-CoV-2 protein–human protein interactions have been experimentally identified, as well as 69 ligands, comprising drugs approved by the US Food and Drug Administration (FDA) and compounds in preclinical and clinical trials, which specifically target these interactions [54]. Understanding which proteins and pathways in the host the virus targets during infection is crucial for the development of AI systems for drug repurposing.

For instance, algorithms modeling the interaction between drugs and proteins have helped identify baricitinib, which was previously used for the treatment of arthritis, as a useful drug against COVID-19 [55]. This drug inhibits the proteins that help the virus penetrate the host cell. Thanks to approaches that exploit the computational identification of relations between existing drugs and target molecules, research published by a team of Korean and American scientists has allowed the identification of FDA-approved antivirals that could potentially target the key proteins for COVID-19 [56].

The molecular processes of virus-host interactions have been recently reconstructed in an international effort coordinated by domain experts, called the COVID-19 Disease Map project [57]. The project aims to maintain an open-access resource for continuous, curated integration of data and knowledge bases to support computational analysis and disease modeling. It represents a milestone of paramount importance for the development of AI systems for SARS-CoV-2 and their comparison with models of other coronaviruses. Moreover, by providing information about the intermolecular wiring of virus-host interactions, the project enables network-based AI modeling for COVID-19 drug repurposing, which has recently shown promising results by using network diffusion and network proximity [58]. Moreover, deep neural networks largely employed in NLP, such as the Transformer architecture, have also been proposed for COVID-19 drug repurposing [56].

In the field of drug development, that is, the pharmacotherapeutic course of a newly identified lead compound, computational models have been proven extremely successful in facilitating a quicker, cheaper, and more effective development of new drugs [59]. For instance, AI can map multidimensional characteristics of proteins to considerably speed up the research process in comparison to traditional methodologies such as x-ray crystallography. In this regard, AI is crucial in optimizing drug discovery pipelines and improving drug development outcomes, with estimated costs of US $2.6 billion [59].

Structural modeling and chemoinformatics methods for COVID-19 (eg, docking-based binding conformation studies of small molecules to target human or viral proteins) can greatly benefit from AI solutions. For instance, AI-based approaches have been used to infer structural similarities among molecules, such as algorithms that can model the graphical structure of chemical compounds through graph convolutional networks or other approaches [60]. AI systems can also leverage knowledge about protein sequences to infer the activity of similar ones. As previously mentioned, Google DeepMind has managed to predict the structure of five proteins targeted by SARS-CoV-2, namely SARS-CoV-2 membrane protein, Nsp2, Nsp4, Nsp6, and papain-like proteinase (C-terminal domain) [4]. The deep learning approach uses amino acid features from similar sequences, based on multiple sequence alignment, to infer the distribution of structural distances to predict the protein structures [61].

Finally, AI can also be used to synthetically generate new molecules, such as new chemical compounds. For instance, the biotech company Insilico Medicine used reinforcement learning to model small molecules and identify those that inhibit specific infection pathways. The team created a generative chemistry pipeline to design novel SARS-CoV-2 inhibitors to later be synthesized and tested. The pipeline employs a large array of generative models, including autoencoders, generative adversarial networks, and genetic algorithms optimized with reinforcement learning [53].

## Pharmacogenomics and Vaccines

Pharmacogenomics, which is the study of the role of genomic characteristics of an individual in drug response, represents a key gateway to personalized medicine [62-64]. Although the translation of genomic information into clinical practice is recognized as one of the most challenging aspects of the future of medicine [65], the information about the genetic makeup of individual patients has the potential to guide clinical decision support and to facilitate biomedical research in many different areas. For instance, genomics can inform drug discovery by providing simultaneous insights into the disease mechanisms and potential targets for treating individual patients [66].

Pharmacogenomics approaches to COVID-19 are still in their infancy. Indeed, although the SARS-CoV-2 genome was published in draft on January 10, 2020 [67], and real-time tracking of the pathogen evolution is now available [68], much less genomic information is currently available about the host. Several studies focus on genetic variations associated with susceptibility to infection and clinical manifestations, including human leukocyte antigen (HLA) variants in the UK Biobank population-based cohort [69] and angiotensin-converting enzyme 2 (ACE2) variants in the Italian population [70]. Retrospective and prospective studies focusing on COVID-19 disease susceptibility and severity have been collected by the COVID-19 Host Genetics Initiative [17,18].

Despite the absence of direct evidence of pharmacogenomics data in COVID-19 patients, the related literature for COVID-19 therapies, including hydroxychloroquine, ribavirin, and baricitinib, has been recently surveyed [71]. Potential actionable genetic markers have been reported, namely, several genetic variants that can alter the pharmacokinetics of drugs that may affect the response to COVID-19 treatments. Importantly, as age, race, gender, and comorbidities have been associated with COVID-19 risk [72], these factors are deemed warranted to assess their role in the variation of treatment responses and need further investigation.

Population genetics is also needed to better understand the association between genetic variability and COVID-19. The importance and complexity of population genetic information, such as genome-wide association studies (GWAS), for drug discovery are exemplified by a study showing that 8% of drugs approved by the FDA target molecules with genetic support, whereas only 2% of phase-1 drugs are genetically supported [73]. Despite such low rates, GWAS can help identify therapeutics that can be repurposed to treat individuals affected by diseases that are mechanistically related to those for which the drugs were developed [74]. Insights from GWAS can also inform about better patient management and therapy, such as the case of variants in six genes on chromosome 3, namely *SLC6A20*, *LZTFL1*, *CCR9*, *FYCO1*, *CXCR6*, and *XCR1*, which have been recently associated with severe COVID-19 cases with respiratory failure [75].

Understanding population genetic heterogeneity is crucial for vaccine design, in particular, as it concerns the individual variability of the major histocompatibility complex (MHC-I and MHC-II) proteins, encoded by the *HLA* gene, which present SARS-CoV-2 epitopes to the immune system. Such individual variability, coupled with the importance of cellular immunity in the severity of the response to the infection, makes the identification of actionable targets for COVID-19 vaccines a challenging endeavor. AI models for COVID-19 vaccine development focus on the prediction of potential epitopes by using a variety of techniques, such as deep docking [76], long short-term memory networks [77], extreme gradient boosting [78], as well as approaches that account for different *HLA* alleles by combining several existing machine learning tools [79]. A recent survey of AI-based approaches to COVID-19 vaccine design [80] suggests that the most popular candidate is the SARS-CoV-2 spike protein, which initiates the interaction with the host through the attachment to the ACE2 receptor [81].

## Mining of the Medical Literature

The staggering rate of publications about COVID-19, both in the form of preprints and peer-reviewed articles, is posing unprecedented challenges to knowledge acquisition and the information quality assessment process. A large part of content is produced by humans for humans, in the form of free text, where crucial pieces of information end up being buried. Because free text is not intelligible by machines, human intervention must identify the relevant pieces of information from the publications and turn it into a tabular form. Recent developments in NLP techniques have helped the automation of this process through machine learning and, in particular, deep learning algorithms [82,83]. Symptoms, patient demographics, clinical data, algorithms, performance, and limitations are identifiable in the texts by properly trained models, which can obtain comparable accuracy to humans at a much faster rate, making it finally possible to monitor the enormous volume of the literature produced [84]. The resulting structured data can be exploited to enrich knowledge graphs (KGs) [85-87], which provide a means to represent and formalize information [85,88], analytical, relational, and inferential investigations and fill the knowledge gaps in the community. Moreover, to rationalize the immense quantity of information on COVID-19, new algorithms can generate low-dimensional representations of the KGs, allowing researchers for clustering and classification [85,89]. We list here representative KG efforts that have been directed at the fight against COVID-19 (see Textbox 1).

**Knowledge graph resources for COVID-19.**

**Project names and references:**

- KG-Covid-19 Knowledge Graph Hub [90]
- COVID-19 Community Project [91]
- COVID-KG [92]
- CovidGraph [93]
- COVID-19 Miner [94]
- COVID-19 Biomedical Knowledge Miner [95]
- COVID-19 Taxila [96]

The KG-Covid-19 Knowledge Graph Hub project is the first Knowledge Graph Hub (KG-Hub) [90] dedicated to COVID-19. KG-Hub is a software to download and transform data to a central location for building KGs from different combinations of data sources. The Covid-19 KG-Hub downloads and transforms data from more than 50 different COVID-19 databases of drugs, genes, proteins, ontologies, diseases, phenotypes, and publications and generates a KG that can be used for machine learning.

The COVID-19 Community Project [91] is a community-based KG that links heterogeneous datasets about COVID-19, in three main areas: the host, the virus, and the cellular environment. These KGs use several publicly available datasets, such as the CORD-19 dataset, a set of over 51,000 scholarly articles about coronaviruses [97].

Other notable databases used in KGs are the COVID-19 Data Portal (see Introduction) and The COVID-19 Drug and Gene Set Library [98]. One of the tools that use these is the COVID-KG [92], which embeds entities in the KG, such as papers, authors, or journals [99].

CovidGraph [93] is a collaboration of researchers to build a research and communication platform that encompasses over 40,000 publications, case statistics, genes and functions, molecular data, and much more. The output is a KG in which entity relationships can be found and new pieces of literature can be discovered. Another tool that uses the CORD-19 dataset is COVID-19 Miner [94], which provides access to a database of interactions among genes or proteins, chemicals, and biological processes related to SARS-CoV-2, which are automatically extracted using NLP from the CORD-19 dataset and manuscripts updated daily from the preprint servers medRxiv and bioRxiv [100].

Furthermore, COVID-19 Biomedical Knowledge Miner [85,95] is an intent to lay the foundation for a comprehensive and interactive KG in the context of COVID-19 that connects the causes and effects and enables users to completely explore the information contained therein. Data are supplied from papers available in PubMed and preprints available from platforms such as bioRxiv, chemRxiv, medRxiv, PrePrints, and Research Square. Lastly, COVID-19 Taxila [96] is an AI and NLP system that uses thousands of COVID-19–related publications, clinical trials, and other relevant sources to enable users to search and analyze the COVID-19 literature. Publications and data are automatically updated.

## Discussion

The COVID-19 pandemic has caused some of the most significant challenges that national health care systems have had to face in recent human history. These systems include not only hospitals but also a multitude of clinicians, retirement and nursing homes, families, and communities. Government lockdown policies undertaken to reduce hospital strain has impacted the society as a whole and has also had social and economic consequences, which have been more severe for minorities and vulnerable groups [101]. Moreover, this pandemic is taking place in the age of social media and Web 2.0, which contain plenty of misinformation and fake news, and with no way for the average internet user to check the reliability of the sources. Nevertheless, the COVID-19 crisis has also shown the promise of technology in facilitating a better understanding of a complex disease and its impact on public health.

Here, we illustrated examples of how AI can advance research and clinical medicine and prepare governments for future similar crises. AI shows promise to deliver models for outbreak analytics and detection, prevention, early intervention, and decision-making. We highlighted the unparalleled opportunity for AI to fill the gap between translational research and clinical medicine. Finally, in addition to the medical applications of AI, it is worth mentioning the potential of NLP for monitoring the quality of the information available to the public and fighting fake news [102-104].

Thanks to the availability of big data and high-performance computing, the fight against the novel coronavirus can leverage the support of AI, as demonstrated by initiatives such as the COVID-19 High Performance Computing Consortium [105]. This technology allows us to address, at a much higher speed and a comparable performance, complex tasks that cannot be executed by humans—who can now focus on more intelligence-demanding activities such as emotional intelligence and human-to-human bonding [106].

Although AI is traditionally trained on large datasets for identifying population-level patterns (ie, common characteristics among people belonging to some clinical classes), recent efforts have promoted the utilization of this technology in conjunction with the principles of precision medicine, to substitute the “average patient” [42] with a real individual, based on geographical and socioeconomic signature as well as genetic, epigenetic, and other molecular profiles [107]. Under this paradigm, AI is meant to empower clinicians to tailor interventions [108] (whether preventive or therapeutic) to the nuanced—and often unique—features of every human being [109]. To this end, multidimensional datasets, such as the variety of data modalities that are currently collected and modeled for COVID-19 [110-112], capture individual genetic, biochemical, physiological, environmental, and behavioral variations [113] that may interfere with the development, progression, and treatment of a disease. Thanks to the drop in price of sequencing the human genome (from billions to hundreds of dollars in 30 years [114]), it is now possible to exploit AI to study phenotypic, genotypic, and environmental correlations among diseases [115]. With this approach, AI can predict the risk of an individual to develop a disease and estimate the likelihood of success for a treatment. In the case of COVID-19, this could lead to a better allocation of resources and an improved match between treatments and patients, consequently improving outcomes for preventive and therapeutic interventions. Therefore, AI-aided precision medicine connects some of the key benefits for a sustainable and effective health care system: efficiency, efficacy, and safety assessment [30].

AI is recognized as a necessity to achieve precision medicine in COVID-19. The current crisis has highlighted that a huge amount of work is still needed to exploit AI-based solutions to their full potential in order to transform health care. AI implementation in the clinical setting is still far from completion [115]. The highly fragmented and diverse health care systems, absence of a protocol for documenting patient data, ethical constraints (such as privacy), and limitations of AI itself (eg, bias and non-interpretability) still represent serious challenges to extensive AI adoption [116].

## Abbreviations

ACE2: angiotensin-converting enzyme 2
AI: artificial intelligence
AUROC: area under the receiver operating characteristic curve
CNN: convolutional neural network
CONSORT-AI: Consolidated Standards of Reporting Trials–Artificial Intelligence
CT: computed tomography
FDA: Food and Drug Administration
GWAS: genome-wide association studies
HLA: human leukocyte antigen
ICU: intensive care unit
KG: knowledge graph
LASSO: least absolute shrinkage and selection operator
NLP: natural language processing
SPIRIT-AI: Standard Protocol Items: Recommendations for Interventional Trials–Artificial Intelligence
